# Intercellular Protein Transfer from Thymocytes to Thymic Epithelial Cells

**DOI:** 10.1371/journal.pone.0152641

**Published:** 2016-03-29

**Authors:** Hong-Xia Wang, Yu-Rong Qiu, Xiao-Ping Zhong

**Affiliations:** 1 Department of Pediatrics, Division of Allergy and Immunology, Duke University Medical Center, Durham, NC, 27710, United States of America; 2 Laboratory Medicine Center, Nanfang Hospital, Southern Medical University, Guangzhou, Guangdong, 510515, China; 3 Department of Immunology, Duke University Medical Center, Durham, NC, 27710, United States of America; 4 Hematologic Malignancies and Cellular Therapies Program, Duke Cancer Institute, Duke University Medical Center, Durham, NC, 27710, United States of America; The Ohio State University, UNITED STATES

## Abstract

Promiscuous expression of tissue restricted antigens (TRAs) in medullary thymic epithelial cells (mTECs) is crucial for negative selection of self-reactive T cells to establish central tolerance. Intercellular transfer of self-peptide-MHC complexes from mTECs to thymic dendritic cells (DCs) allows DCs to acquire TRAs, which in turn contributes to negative selection and regulatory T cell generation. However, mTECs are unlikely to express all TRAs, such as immunoglobulins generated only in B cells after somatic recombination, hyper-mutation, or class-switches. We report here that both mTECs and cortical TECs can efficiently acquire not only cell surface but also intracellular proteins from thymocytes. This reveals a previously unappreciated intercellular sharing of molecules from thymocytes to TECs, which may broaden the TRA inventory in mTECs for establishing a full spectrum of central tolerance.

## Introduction

Proper intrathymic T cell development ensures the generation of a repertoire of T cells against various pathogens but also self-tolerant. Thymus is composed of multiple cell lineages of different origins, such as developing T cells, dendritic cells (DCs), macrophages, B cells, and thymic epithelial cells (TECs). The thymus is separated into the cortex and medulla, which are involved in the distinct function of the thymus with regard to T cell development [1–3]. Early thymic progenitors enter the thymus at the conjunction between medulla and cortex. These cells, phenotypically CD4^-^CD8^-^ double negative (DN), migrate toward the cortex to initiate early T cell development [4]. After successful recombination of the T cell receptor β gene and expression of the pre-TCRα/β receptor, these cells mature to the CD4^+^CD8^+^ double positive (DP) stage, at which the TCRα gene rearranges [5]. Expression of a functional αβ TCR on DP thymocytes and engagement of these TCRs with self-peptide major histocompatibility complex (MHC) expression on cortical TECs (cTECs) ensures their survival and differentiation to the CD4^+^CD8^-^ and CD4^-^CD8^+^ single positive (SP) stage, also known as positive selection. SP thymocytes migrate into the medulla, where they engage with medullary TECs (mTECs) and DCs via TCR and self-peptide MHC interactions [1]. SP thymocytes expressing TCRs with high affinities to self-peptide–MHC complexes are self-reactive and are eliminated from the T cell repertoire due to programmed cell death, a process also called negative selection for establishing central tolerance. SP thymocytes with weak affinities to self-peptide–MHC complexes escape negative selection for populating peripheral lymphoid organs [6].

To establish central tolerance, mTECs must express tissue-restricted antigens (TRAs), which requires the transcription factor Aire [7–11]. Deficiency of Aire causes defective TRA expression, impaired mTECs maturation, and severe autoimmune diseases in both mice and humans [7, 12]. Besides directly triggering negative selection, mTECs share the burden with medullar DCs to establish central tolerance [13, 14]. Although DCs do not actively transcribe *TRAs*, they can acquire TRAs and self-peptide–MHC complexes from mTECs via intercellular protein transfer. Thymic DCs have been found to play important roles in negative selection of self-reactive T cells as well as for induction of regulatory T cells via acquisition of TRAs and MHCs from mTECs [15–19]. Although Aire has the capacity to induce promiscuous transcription of *TRAs* in mTECs, it is hard to envision that all *TRAs* are actively transcribed in mTECs. Furthermore, some TRAs can only be generated after somatic recombination events that are strictly tissue/cell lineage specific, such as TCRs and immunoglobulins in thymocytes/T cells and B cells, respectively. Additional mechanisms must exist for mTECs and DCs to acquire TRAs. We report here that not only cell surface but also intracellular proteins can be efficiently transferred from thymocytes to both mTECs and cTECs, revealing a novel mechanism for mTECs to acquire thymocyte TRAs via intercellular transfer.

## Materials and Methods

### Ethics Statement

This study was carried out in strict accordance with the recommendations in the Guide for the Care and Use of Laboratory Animals of the National Institutes of Health. Experiments in this study were performed according to protocols (A095-13-04) approved by the Institutional Animal Care and Usage Committee of Duke University.

### Mice

C57BL6/J, *Rosa26-LSL-ZsGreen* [20], *Rosa26-LSL-Tdtomato* [20], and *TCRα*^*-/-*^ [21] mice were purchased from the Jackson laboratory. *CD4Cre* mice [22] were purchased from Taconic Inc. *Foxn1Cre* mice [23] were kindly provided by Dr. Nancy Manley, University of Georgia. The mice were housed in a pathogen-free facility and were bred as described in the Results section. Mice were euthanized by CO2 followed by organ removal. Total 40 mice (18 male and 22 female mice) were used for experiments.

### Antibodies and Flow Cytometry

The following antibodies used for flow cytometry were purchased from Biolegend: anti-CD4 (clone GK1.5), CD8 (clone 53–6.7), CD45 (clone 30-F11), CD45.2 (clone 104), EpCAM/CD326 (clone G8.8), Ly51 (clone 6C3), IgG isotype control, Ulex Europaeus Agglutinin I (UEA-1, clone B-1065; vector laboratories). Cells were stained for surface molecules using 2% FBS-PBS as previously described [24]. Cell death was identified by 7-AAD staining. Stained samples were acquired on a FACS Canto-II (BD Biosciences) flow cytometer. Data were analyzed with FlowJo software (Tree Star) and were gated on live cells and singlets.

### Preparation of TEC single cell suspension

TEC single cell preparation was performed according to published protocols with modifications [25, 26]. Thymi were gently removed and trimmed of fat and connective tissue in cold RPMI-1640 containing 2% FBS. The thymus was then cut into small pieces (<2mm), which were suspended in 2ml digestion buffer containing 250μl collagenase type IV(10mg/ml; Worthington), 40μl DNase (50mg/ml; Worthington), and 1.7ml free-FBS RPMI-1640 shaking at 150–200 rpm at 37°C in an incubator for 8–10 min. Digested thymus remnants were settled at room temperature for 1 minute, and supernatants were transferred to new tubes. The remaining thymus fragments were digested three additional times. After digestion, combined samples were spun down at 472g for 5min. Cell pellets were resuspended in IMDM-10, washed two times by centrifugation, and eventually resuspended in cold EDTA/FACS buffer (5Mm EDTA, 2%FBS in PBS). Cells were immediately used for cell surface and intracellular staining with indicated antibodies.

### Bone Marrow Chimeras

Bone marrow (BM) cells, isolated from *Rosa-LSL-ZsGreen* and *Rosa-LSL-ZsGreen-CD4Cre* donor mice, were depleted of T cells with a PE conjugated anti-CD3 antibody (Biolegend) and anti-PE microbeads (Miltenyi Biotec) according to the manufacturer’s protocol. C57BL/6J recipient mice were lethally irradiated (1000 rad) and intravenously injected with 15 x 10^6^ BM cells. After reconstitution, mice were monitored for movement, fur color, and weight daily in the first two weeks and every other day afterwards. Mice with weight loss greater than 15% would be euthanized according to our approved protocol. All mice in these experiments were healthy prior to the experimental endpoint. One month after BM reconstitution, single cell suspensions from the thymus were prepared and stained for flow cytometry analysis.

### Immunofluorescence microscopy

Thymus lobes were embedded in OCT (Leica Biosystems Richmond Inc.) and frozen immediately at -80°C. Frozen thin sections (5μm) were cut and fixed in a 1:1 mixture of acetone and methanol at -20°C for 8 minutes. Sections were air-dried and kept at -20°C. After being warmed up to room temperature (RT), the frozen sections were blocked with PBS containing 3% BSA and 0.1% Triton X-100 for 30–45 minutes at room temperature, stained with primary rat anti-mouse keratin 8 (KRT8, Troma-1, DSHB, University of Iowa; 1:50 dilution) or rabbit anti-mouse KRT5 (PRB-160P, Covance; 1:200 dilution), and finally stained with a secondary Rhodamine-conjugated donkey anti-rabbit IgG antibody (1:400 dilution) or Rhodamine-conjugated goat anti-rat IgG antibody (Jackson ImmunoResearch Laboratories Inc.; 1:300 dilution). After staining, samples were mounted with Vector mounting solution containing DAPI (Vector) and allowed to dry overnight at RT in the dark. Images were acquired using a Zeiss ApoTome Microscope and analyzed using Photoshop CS6 software.

## Results

### Expression of hematopoietic/thymocyte specific molecules in both cTECs and mTECs

**Fig 1:**
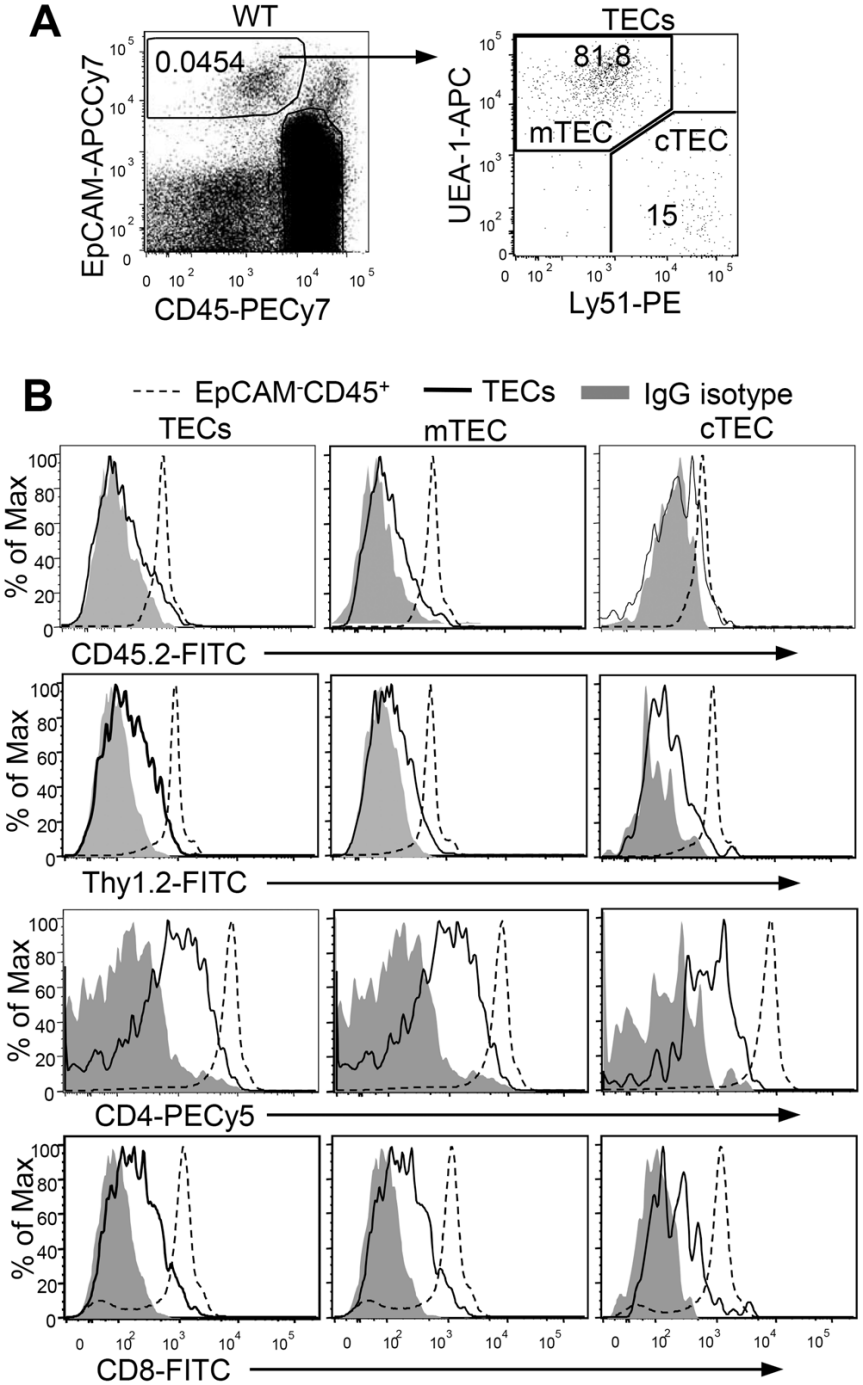
Detection of thymocyte/hematopoietic specific cell surface markers in TECs. **A.** Representative dot-plot of CD45 (PECy7) and EpCAM (APCCy7) staining of single cell suspension of thymus and UEA-1 (Biotin-strepavdin-APC) plus Ly51 (PE) staining in live and singlet gated CD45^low^EpCAM^+^ TECs in WT thymocytes. **B.** Overlaid histogram for CD45.2 (FITC), Thy1.2 (FITC), CD4 (PECy5), and CD8 (FITC) or IgG isotype control staining in gated TECs (CD45^low^EpCAM^+^), mTECs (CD45^low^EpCAM^+^UEA-1^+^Ly51^-^), cTECs (CD45^low^EpCAM^+^UEA-1^-^Ly51^+^), and CD45^+^EpCAM^-^ thymocytes. Data shown are representative of at least three experiments.

Cells derived from hematopoietic stem cells express the protein tyrosine phosphatase CD45. In contrast, TECs are phenotypically defined as CD45^-^EpCAM^+^ [27]. When analyzing TECs from wild-type (WT) mice, we noted that the traditionally defined CD45^-^EpCAM^+^ TEC population actually positioned as CD45^low^ (Fig 1A). To rule out the possibility that an intermediate level of CD45 expression detected in TECs by FACS analysis was due to autofluorescence of this population, we stained TECs simultaneously with a PECy7-labeled anti-CD45 and a FITC-labeled anti-CD45.2 or a FITC-labeled IgG isotype control. TECs, gated as CD45^low^EpCAM^+^, were examined for CD45.2 or isotype control staining. As shown in Fig 1B, CD45.2 was detected in TECs at levels above isotype control staining, indicating that CD45 was indeed expressed on TECs, although it was expressed at levels lower than cells of the hematopoietic origin (CD45^+^EpCAM^-^). Further analysis revealed that both mTECs (UEA-1^+^Ly51^-^) and cTECs (UEA-1^-^Ly51^+^) expressed low levels of CD45.2 (Fig 1B). Moreover, other T cell specific markers, such as Thy1.2, CD4, and CD8, were also weakly detected in total TECs and m/cTECs. Together, these observations revealed that TECs appear to promiscuously express low levels of thymocytes/hematopoietic specific molecules.

### Transfer of intracellular fluorescent proteins from thymocytes to cTECs and mTECs

**Fig 2:**
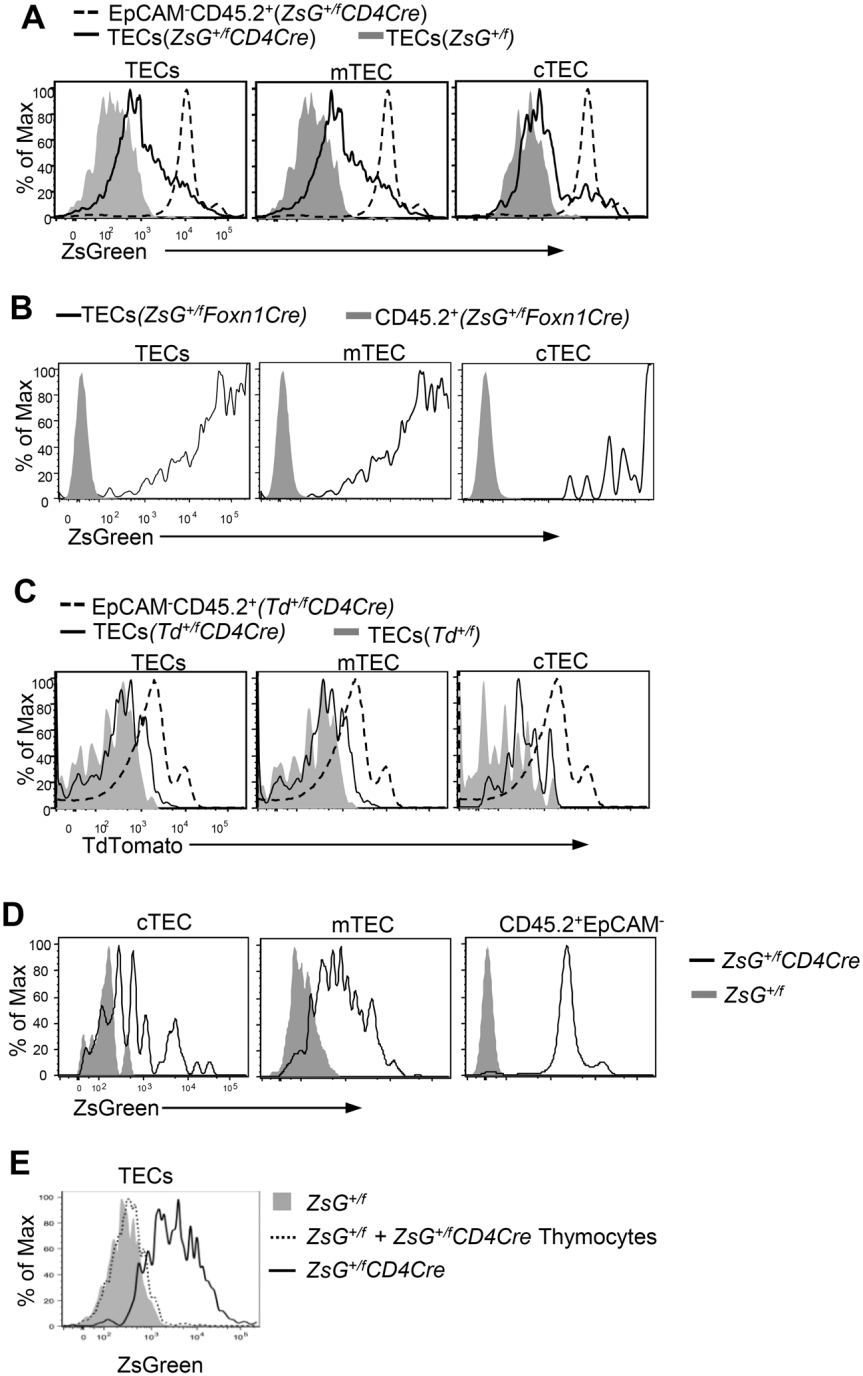
Intercellular transfer of intracellular fluorescent proteins from thymocytes to TECs. **A.** Overlaid histogram for ZsGreen intensity in gated TECs (CD45.2^low^EpCAM^+^), mTECs (CD45.2^low^EpCAM^+^UEA-1^+^Ly51^-^), cTECs (CD45.2^low^EpCAM^+^UEA-1^-^Ly51^+^), and CD45^+^EpCAM^-^ and CD45.2^+^EpCAM^-^ thymocytes from *Rosa-LSL-ZsGreen-CD4Cre* mice and *Rosa-LSL-ZsGreen* control mice. **B.** Overlaid histogram for ZsGreen intensity in gated TECs (CD45.2^low^EpCAM^+^), mTECs (CD45.2^low^EpCAM^+^UEA-1^+^Ly51^-^), and cTECs (CD45.2^low^EpCAM^+^UEA-1^-^Ly51^+^), and CD45.2^+^EpCAM^-^ thymocytes from *Rosa-LSL-ZsGreen-Foxn1Cre* mice and *Rosa-LSL-ZsGreen* control mice. **C.** Overlaid histogram for TdTomato intensity in gated TECs (CD45.2^low^EpCAM^+^), mTECs (CD45.2^low^EpCAM^+^UEA-1^+^Ly51^-^), and cTECs (CD45.2^low^EpCAM^+^UEA-1^-^Ly51^+^), and CD45.2^+^EpCAM^-^ thymocytes from *Rosa-LSL-TdTomato-CD4Cre* mice and *Rosa-LSL-TdTomato* control mice. **D.** Detection of ZsGreen in TECs from irradiation chimeric C57BL/6J mice reconstituted with BM cells from *Rosa-LSL-ZsGreen-CD4Cre* mice. C57BL6/J mice were lethally irradiated (1000 rad) and reconstituted with BM cells from *Rosa-LSL-ZsGreen-CD4Cre* mice or *Rosa-LSL-ZsGreen* control mice. Thirty days after transfer, TECs from recipients were examined for ZsGreen intensity. Overlaid histogram shows ZsGreen intensity in gated mTECs (CD45.2^low^EpCAM^+^UEA-1^+^Ly51^-^), cTECs (CD45.2^low^EpCAM^+^UEA-1^-^Ly51^+^), and CD45.2^+^EpCAM^-^ thymocytes from recipient mice. **E.** ZsGreen^+^ thymocytes were unable to transfer ZsGreen to TECs during *ex vivo* TEC preparation. Total thymocytes from *Rosa-LSL-ZsGreen-CD4Cre* mice were pelleted and resuspended in 50 μl digestion buffer and transferred to *Rosa-LSL-ZsGreen* thymus during TEC preparation, starting at the tearing of the thymus. The single cell suspension was similarly stained as in Fig 1. Overlaid histograms show ZsGreen intensity in TECs from *Rosa-LSL-ZsGreen* thymus, whether or not mixed with ZsGreen^+^ thymocytes. TECs from *Rosa-LSL-ZsGreen-CD4Cre* mice were used as positive controls. Data shown are representative of three (A–D) and two (E) experiments with at least one pair of mice in each experiment.

Low level expression of thymocyte-specific molecules in TECs, particularly in mTECs, could be the result of promiscuous transcription of these molecules in TECs. To rule out this possibility and to further examine whether TECs could acquire molecules inside thymocytes, we bred the *Rosa-LSL-ZsGreen* reporter mice [20] with mice carrying the *CD4Cre* transgene, which mediates T cell specific deletion of gene segments flanked by two *loxp* sites [22]. Deletion of the *Loxp-STOP-Loxp* cassette inserted upstream of the ZsGreen gene allows high level expression of ZsGreen protein in CD45^+^EpCAM^-^ thymocyte (Fig 2A). More important, low level ZsGreen could also be detected in total TECs as well as cTECs and mTECs.

To rule out that low levels of ZsGreen expression in the TECs were not caused by Cre-mediated deletion of the *Loxp-STOP-Loxp* cassette in these cells, we examined the same *Rosa-LSL-ZsGreen* reporter mice carrying the *Foxn1Cre* transgene [23], which mediates TEC-specific deletion of the *Loxp-STOP-Loxp* cassette. ZsGreen in TECs from *Rosa-LSL-ZsGreen-Foxn1Cre* mice was directly under the control of the *Rasa26* promoter, and the actin promoter knocked into the locus. As shown in Fig 2B, ZsGreen level in these TECs was much higher than that detected in *Rosa-LSL-ZsGreen-CD4Cre* mice. Such thymocyte to TEC transfer of intracellular proteins was not limited to ZsGreen. TECs from *CD4Cre* mice carrying a conditional Tdtomato reporter, *Rosa26-LSL-TdTommato* [20], were also detected to weakly express Tdtomato (Fig 2C). Together, these observations revealed that intracellular proteins in the thymocytes can be transferred into TECs.

Although data from *Rosa-LSL-ZsGreen-CD4Cre* mice supported that intercellular transfer of proteins from thymocytes to TECs occurred in the thymus, they did not firmly rule out that active transcription and translation of low levels of ZsGreen in TECs in *Rosa-LSL-ZsGreen-CD4Cre* mice. To address this issue, we generated irradiation chimeric mice using BM cells from *Rosa-LSL-ZsGreen-CD4Cre* mice. One month after reconstitution, we detected not only high levels of ZsGreen expression in the thymocytes, but also significant ZsGreen expression in both mTECs and cTECs (Fig 2D), ruling out that ZsGreen expression in TECs in these mice was resulted from low levels of ZsGreen transcription and translation in these cells.

To further rule out that the ZsGreen detected in TECs from *Rosa-LSL-ZsGreen-CD4Cre* mice by flow-cytometry was an artifact introduced during *ex vivo* processing of the thymus such as formation of TEC—thymocyte conjugates or uptake of Zsgreen protein released from thymocytes, we first isolated total thymocytes from *Rosa-LSL-ZsGreen-CD4Cre* mice and then mixed these ZsGreen^+^ thymocytes in high density with *Rosa-LSL-ZsGreen* thymus during TEC preparation. As shown in Fig 2E, ZsGreen intensity in TECs from the preparation mixed with ZsGreen^+^ thymocytes was similar to that in TECs without mixed with ZsGreen^+^ thymocytes, suggesting that *ex vivo* preparation of TECs was not sufficient to transfer ZsGreen from thymocytes to TECs.

### Impact of TCR-MHC engagement on protein transfer from thymocytes to TECs

**Fig 3:**
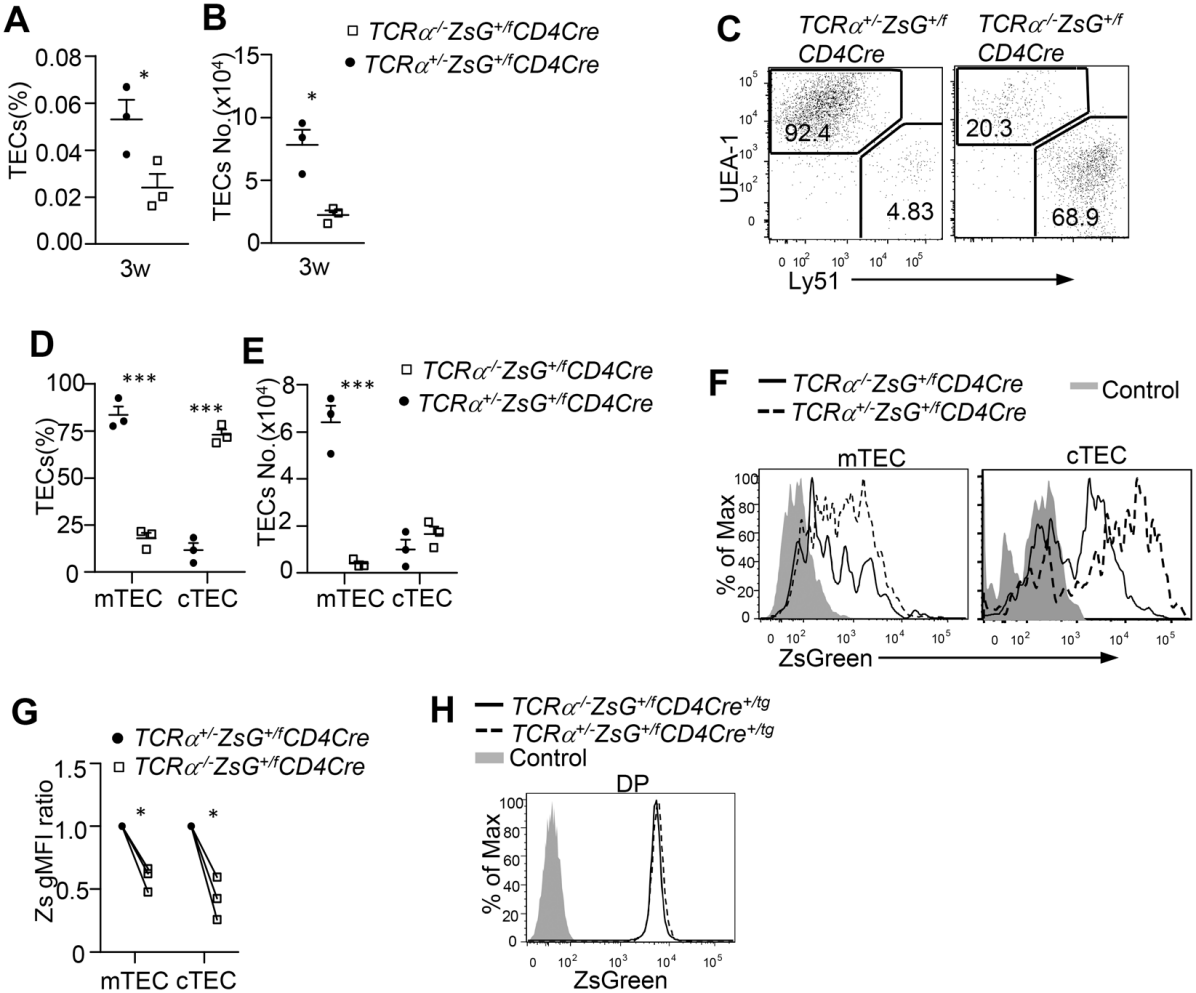
Contribution of TCR-MHC engagement dependent and independent mechanisms to protein transfer from thymocytes to TECs. **A, B.** Frequencies (A) and absolute numbers (B) of TECs in *TCRα*^*-/-*^*ZsGreeen-CD4Cre* and *TCRα*^*+/-*^*ZsGreeen-CD4Cre* mice. **C.** Representative dot plots of showing mTEC and cTEC subsets. **D, E.** Percentages (D) and absolute numbers (E) of mTECs and cTEC. **F.** Overlaid histograms showing ZsGreen level in mTECs and cTECs. **G.** Relative gMFI of ZsGreen in m/cTECs. gMFI in *TCRα*^*+/-*^*ZsGreeen*^*f/+*^*CD4Cre* TECs was arbitrarily set as 1. **H.** ZsGreen intensity of CD45^+^EpCAM^-^CD4^+^CD8^+^ DP thymocytes. Data shown are representative or calculated from three experiments. *, *p*<0.05 determined by Student *t*-test. Raw data for Fig 3A, 3B, 3D, 3E, and 3G are shown in S1 Data.

Developing thymocytes engage with self-peptide–MHC complexes expressed on cTECs and mTECs via TCRs to mediated positive and negative selection, respectively. To examine whether such engagement may affect protein transfer from the thymocytes to TECs, we analyzed *TCRα*^*-/-*^*ZsGreeen-CD4Cre* and *TCRα*^*+/-*^*ZsGreeen-CD4Cre* mice. Similar to previously reported [21], SP thymocytes were virtually absent in *TCRα*^*-/-*^*ZsGreeen-CD4Cre* mice, but not in *TCRα*^*+/-*^*ZsGreeen-CD4Cre* mice. Crosstalk between SP thymocytes and TECs is important for mTEC maturation and survival [28–32]. TEC percentages and total TEC numbers were decreased in *TCRα*^*-/-*^*ZsGreeen-CD4Cre* mice (Fig 3A and 3B), accompanying obvious decreases of mTEC percentages and numbers (Fig 3C–3E). Although cTEC percentages were relatively increased, cTEC numbers were not noticeably changed in *TCRα*^*-/-*^*ZsGreeen-CD4Cre* mice compared with *TCRα*^*+/-*^*ZsGreeen-CD4Cre* mice. Importantly, ZsGreen intensity in both mTECs and cTECs from *TCRα*^*-/-*^*ZsGreeen-CD4Cre* mice were lower than those from *TCRα*^*+/-*^*ZsGreeen-CD4Cre* mice (Fig 3F and 3G). Because DP thymocytes from both mice expressed similar levels of ZsGreen (Fig 3H), it suggested that decreased ZsGreeen intensity in *TCRα*^*-/-*^*ZsGreeen-CD4Cre* TECs was not caused by abnormal ZsGreen expression in thymocytes from these mice. Thus, engagement between TECs and thymocytes via TCR-MHC interactions might positively contributed to protein transfer from thymocytes to TECs. Alternatively, TCR-MHC interaction could promote protein uptake by mTECs through promoting mTEC maturation. Because Zsgreen levels in TECs from *TCRα*^*-/-*^*ZsGreeen-CD4Cre* mice were still higher than those in TECs from ZsGreen negative control mice, this data suggested that TCR-MHC independent mechanism(s) also contributed to protein transfer from thymocytes to TECs.

## Discussion

Here, we provide the first evidence that mTECs and cTECs are capable of acquisition of both cell surface and intracellular proteins from thymocytes. Because TCRα deficiency considerably decreased ZsGreen acquisition by TECs from thymocytes, TCR-MHC engagement-dependent mechanisms must be involved in intercellular protein transfer from thymocytes to TECs. However, we cannot rule out whether third parties such as DCs and macrophages are also involved in the transfer from thymocyte to TECs. Additionally, our data suggests that TCR independent transfer of protein from thymocyte to TECs occurs. At present, how proteins are transferred from thymocytes to TECs remains unknown, but the process is likely involved in multiple mechanisms. Trogocytosis, which transfers membrane patches and associated proteins through “membrane nibbling,” nanotubes connecting different cells, and gap junctions have been reported to be involved in intercellular protein transfer [33–35]. Thymocytes can also release exomes [36, 37], which could be uptaken by TECs. Additionally, apoptotic thymocytes could be uptaken by DCs and macrophages or directly by TECs as well as through release of thymocyte specific components in local environment and subsequently uptaken by TECs. In support of this possibility, we have found that TECs are able to uptake soluble proteins in vitro and process uptaken proteins in an acidic compartment (Data not shown).

Previous reports have demonstrated intercellular transfer of proteins from mTECs to thymic DCs [13, 14]. Unidirectional transfer of self-peptide–MHC complexes from mTECs to DCs allows DCs to present self-TRAs to SP thymocytes for induction of negative selection and generation of regulatory T cells[15–19]. Although the physiological importance of protein transfer from thymocytes to TECs remains to be illuminated, it is conceivable that such transfer may broaden TRA inventory in mTECs by inclusion of antigens that are normally only expressed in thymocytes but not transcribed in mTECs. For example, TCRs are only generated after somatic V(D)J recombination that occurs strictly in thymocytes. Transfer of TCRs and other T cell specific proteins from thymocytes to TECs may provide a pathway to allow T cell–specific antigens to be presented by TECs, particularly mTECs, which may induce negative selection of self-reactive T cells against T cell–restricted antigens such as TCRs. Interestingly, a recent study has found that intrathymic B cells are licensed to present TRAs to induce negative selection [38]. It would be interesting to determine whether thymic B cells may uniquely present immunoglobulin epitopes to prevent generation of B cell–reactive T cells.

## Supporting Information

S1 Data Raw data for analyses shown in Fig 3 of the manuscript. (XLSX)
